# Supplementary material for: Assessment of the Arabic patient-centered online information about orthodontic pain: A quality and readability assessment
Source: PLoS One. 2024 May 23;19(5):e0303308. doi: 10.1371/journal.pone.0303308 (PMC11115317; doi:10.1371/journal.pone.0303308)
Supplement: Attachment — Submitted filename: Responses to Reviewers.docx [file pone.0303308.s002.docx]

**Response to Reviewer 1**

1. **Affiliation Correction**: The letter “n” at the end of the word “Arabia” in the affiliation of the first author has been omitted as requested.
2. **Abstract Structure**: The abstract has been revised to be structured clearly into sections corresponding to the background, materials and methods, results, and conclusion.
3. **Introduction Formatting**: A space has been added after the verb “peaks” in the third paragraph of the introduction as suggested.
4. **Reference Citation**: Reference 20 is now cited only once at the end of the fourth paragraph on page 4, addressing the concern of repetitive citation.
5. **Reliability Assessment**: We have clarified the reliability assessment process in the revised manuscript.
6. **Readability Assessment Tools**: An explanation regarding the validation of readability assessment tools for Arabic texts has been included in the revised manuscript. Our decision not to conduct a separate validation process for these tools in the context of Arabic texts was informed by precedent. Previous research has applied these evaluation tools across a variety of languages without specific validation for each linguistic context. This approach is based on the assumption that the criteria these tools assess—such as accuracy, transparency, and evidence-based content—are universal in nature and not dependent on language.
7. **DISCERN Assessment Scores**: The error in referencing Table 2 instead of Table 4 has been corrected.
8. **Table 3 Correction**: The description of Table 3 in the text has been revised to accurately include and reflect the readability scores of the websites alongside the reliability scores, rectifying the previous oversight.
9. **Tables Improvement**:
   - The typo “zs” next to Q2 in Table 2 has been corrected.
   - Interquartile values have been added to Table 2.
   - We have resolved the critical inconsistency between the "mean" and "median" descriptions in Tables 3 and 4, ensuring that all descriptions accurately match the presented data.
10. **JAMA Benchmarks Clarification**: That is true for commercial websites, but authorship is the second most common for all websites, as stated in the total column.
11. **Alignment of Descriptions and Tables**: The manuscript has been thoroughly reviewed to ensure that all points, especially 7, 8, 9, and 10, are correctly aligned with their associated tables, and any contradictory results have been addressed and organized appropriately.
12. **List of Abbreviations**: A list of abbreviations has been included at the end of the manuscript, providing clear definitions for all assessment tools mentioned throughout the text.

**Response to Reviewer 2**

1. **Study Type**: The study is retrospective, as clarified in the revised manuscript's materials and methods section.
2. **Bias Prevention**: The revised manuscript now clearly states that to prevent search biases, searches were conducted in incognito mode with search history and cookies erased before the searches on October 2023.
3. **Search Date**: The date of the website search, October 2023, has been explicitly stated in the materials and methods section.
4. **Validity of Tools**: We have added a section in the methodology explaining why the validity of the JAMA, HON, and DISCERN tools was not tested specifically for Arabic texts. This decision was based on the precedent of these tools' universal application across languages without specific linguistic validation.
5. **Referenced Study**: The specific study referenced regarding the discomfort experienced by lower incisors has now been cited correctly in the manuscript.
6. **Reference Addition**: References have been added after each requested sentence, ensuring that all claims are adequately supported by the literature.
7. **Reference Update**: Older references, particularly those published prior to 2000, have been reviewed and updated where appropriate to reflect more current research findings in the field, except for some studies as needed.
